# Defining Anti-arrhythmic Efficacy in the Setting of Evolving Concepts About Atrial Fibrillation

**DOI:** 10.19102/icrm.2023.14016

**Published:** 2023-01-15

**Authors:** James A. Reiffel

**Affiliations:** ^1^Columbia University, New York, NY, USA

**Keywords:** Anti-arrhythmic drugs, atrial fibrillation, efficacy, rhythm control, quality of life

As the Pharmacologic Insights Section Editor of *The Journal of Innovations in Cardiac Rhythm Management*, I have the opportunity each year to write an end-of-year commentary for this section if one seems appropriate. It might be about new rhythm-related pharmacologic agents that were released during the past year, new updated or revised information about rhythm-management drugs based upon recent trial results or new guidelines, or new or evolving concepts underlying pharmacologic therapy of ≥1 rhythm abnormalities, etc. In the year that just ended, no new anti-arrhythmic or anticoagulant agents became available. Moreover, reported major arrhythmia trials, such as those for atrial fibrillation (AF), did not provide any strikingly new information about rhythm-management pharmaceuticals (aside from yet more ablation vs. anti-arrhythmic drug [AAD] comparisons for which multiple reviews are already available). An excellent review of possible future drug therapies for AF has also recently been published.^1^ With this in mind, for 2022, I have elected to comment upon a consideration related to all of the above—efficacy endpoints for pharmacologic (or ablative) AF rhythm control—including their definition, their application to clinical practice, and subtleties regarding their interpretation from clinical trials.

## What defines efficacy?

The reasons to treat any disorder, including AF, are to allow a patient to live longer and/or to improve their quality of life (QoL). The latter also involves minimizing adverse effects consequent to the therapy beyond improving the QoL associated with the disorder being treated. However, what does this mean with respect to AF?

A 72-year-old woman with a history of well-controlled hypertension on an angiotensin-converting enzyme inhibitor, well-controlled hyperlipidemia on a statin, and a 4-year history of paroxysmal AF has been managed so far with carvedilol and apixaban. Initially, her episodes were quite brief (minutes to an hour or so) and infrequent (every 6–10 months). However, in the past year, they have increased in frequency to several per month and now last up to 60 h, with 1 episode requiring direct current cardioversion. When she is in sinus rhythm, she feels well and can do all routine activities, play golf (walking the course), and enjoy time with her grandchildren. When she is in AF, she feels somewhat fatigued, has mild-to-moderate dyspnea when walking up hills during golf, and has felt slightly dizzy once or twice at the onset of an episode. Her ventricular rate during an event-monitored episode was in the 80s at rest. She has now been referred to you for rhythm control. After discussing the options with the patient, you elect to begin ≥1 AAD trial before considering ablation.

How would you define AAD efficacy in this patient’s case? How would she define it? Is efficacy defined by prolonging the time to her first episode of recurrent AF on the AAD? This is an endpoint that has most commonly been used to define efficacy in clinical trials.^2^ Or, is efficacy better defined by reducing the total amount of AF suffered by the patient—that is, the AF burden (AFB), i.e., the percentage of time spent in AF?^1^ AFB relates to both the number of AF events and their duration over defined periods of time (such as 1, 2, or 5 years). Unfortunately, accurate assessment of AFB requires continuous monitoring of cardiac rhythm—for at least a representative period of time—which is best done using an inserted cardiac monitor (ICM). External devices, including smartwatches, loop recorders, and the like, do not record continuously or for long enough periods to accurately quantitate AFB, and monitoring with intermittent electrocardiograms or periodic Holter recordings is even more problematic with respect to AFB. Also, unfortunately, most clinical trials, including the ablation versus AAD trials, performed over the past 2 decades have not used ICMs; thus, the accuracy of their assessments may be imprecise (especially if intermittent and/or asymptomatic periods of AF were not recognized). Lastly, would this patient define treatment efficacy by an absolute amount of AFB reduction or by an improvement in symptoms with satisfactorily fewer and shorter total events?

For symptomatic AF, as found in this patient’s case (in contrast to asymptomatic events), it is the AFB and adequate symptom reduction, not just the time to first recurrence, that will relate to a patient’s QoL and their satisfaction or dissatisfaction with the therapy chosen. Importantly, the time to first recurrence following the onset of therapy is not highly correlated with improvements in overall AFB during a course of therapy.^2^ Also important is the fact that, in AF patients, the presence of symptoms does not reliably correlate with the presence of AF.^2,3^ Thus, each patient needs to be questioned and assessed carefully.

**Figure 1:**
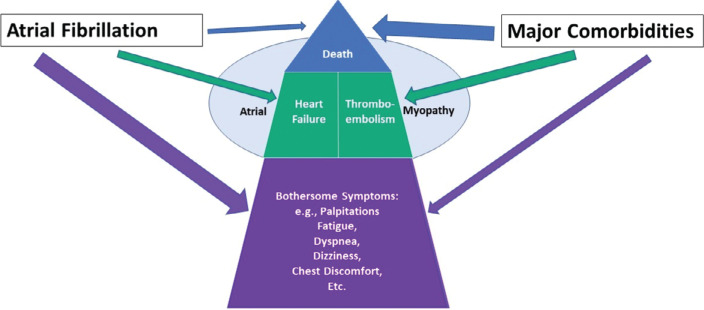
A schematic diagram showing the relative frequency (indicated by the height of each section in the diagram) of symptoms (highest) versus non-fatal major outcomes (such as heart failure or thromboembolism) versus mortality (least frequent). The relative importance of the contributing factors (atrial fibrillation and major comorbidities) to each section of the diagram is indicated by the width of the arrows, with wider arrows indicating a greater contributing effect and narrower arrows indicating less of an effect or the least effect.

Clearly, the reduction of total symptoms during the course of a patient’s care is more important than simply prolonging the time to the first recurrent event. However, is this an adequate definition of efficacy? Certainly, it is not the whole story. What about the prevention/reduction of the risk of a major adverse outcome event, such as heart failure (HF), a thromboembolic event (TE), hospitalization, or death, in a patient with AF?^2^ The prevention of major adverse outcomes must be considered beyond simply reducing AF-associated symptoms, and such are of concern both in patients with episodes of symptomatic AF as well as in those whose AF remains asymptomatic until a major adverse outcome brings it to attention. Moreover, outcomes such as HF, TE, hospitalization, and death have been used as efficacy-defining events in recent clinical trials (as I noted in my 2021 year-end commentary^4^) as they are easy to count, rather non-subjective, and in that respect are considered useful for U.S. Food and Drug Administration (FDA) analyses, similar to the time to first recurrence. These contrast with qualitative but not quantitative efficacy criteria, such as QoL.^2^ Of further importance, primary outcomes in clinical trials are most often assessed by intention-to-treat statistics. Confoundingly for the clinician, in actual fact, there is often crossover between limbs in clinical trials. Additionally, ancillary treatments may also be employed. For example, in the ablation arms of recent ablation versus AAD trials for AF, up to 50% of the ablation patients were also given AADs during their post-ablation care.^5–7^ Thus, are the frequent reports that ablation is superior to AADs for AF reduction accurately forthcoming? Some observers say no, as they often include hybrid treatment using ablation plus AADs versus AADs alone—data that are usually found “in the fine print” but not included in the report’s abstract. Relevant to clinical practice, if a certain therapy is not adequate, practitioners commonly change treatments or try combinations of therapy (including AADs post-ablation). We are not limited to ablation versus AADs. Also, of relevance to clinical practice, both AF itself and underlying comorbidities do not contribute equally to symptoms, HF, hospitalizations, or mortality. Mortality, for example, is, to a large extent, a function of comorbidity presence, number, and severity, rather than the rhythm itself, whereas symptoms are often notably worse in the presence of AF or are directly mediated by AF (see **Figure 1**). Thus, discussions with a patient about efficacy regarding AAD (or ablative) therapy need to consider what is being measured and on what background their AF is occurring.

Accordingly, defining the efficacy of rhythm-control therapy in AF is more complex in practice than it might appear at first consideration, and, in the “real world,” therapeutic effectiveness must consider improvements in QoL, the reduction of major adverse outcomes when efficacy is being assessed, and whether the therapy reportedly tested in a clinical trial is really the therapy that was used. If a therapy were to be associated with a reduction in AFB and AF-associated symptoms, but the patient had a major debilitating stroke, would the treating physician really believe their treatment had been adequate? Conversely, if a patient had some brief, well-tolerated, infrequent recurrences, but their overall QoL was substantially improved on the chosen AAD regimen, might not the patient feel that therapeutic efficacy had been attained? If an AF patient never had an HF or TE event, would they focus upon this absence, rather than their improved QoL, to define the efficacy of a rhythm-control therapy? Likely not, in contrast to a patient who had already suffered such a major adverse outcome event.

My purpose in discussing the above is to enhance the reader’s awareness regarding the interpretation of the results of published trials on AF therapy more adequately as well as to enhance the use of trial results in the planning of care for one’s own patients. When assessing AF trial-reported outcomes, the astute reader needs to understand the limitations and biases of the endpoints used to define efficacy in each trial. If time to first recurrence is used, ask yourself if that is really relevant to you (in contrast to FDA historical practices). If the number of AF recurrences or AFB is used, ask yourself how it was measured, recalling that non-continuous recordings are not definitive or quantitatively accurate. If symptoms were considered, recognize that they may or may not correlate with recurrences of AF. If major adverse outcome composites are the primary events defining efficacy, ask yourself if they are applicable to the specific patient sitting across from you when you and they decide on a therapeutic rhythm-control plan. For additional consideration of these patient-care versus clinical trial issues, please see the article by Reiffel and Naccarelli.^8^

## An additional consideration regarding the efficacy of therapy for atrial fibrillation— timing must also be considered

In the early 2000s, it was observed, but not adequately appreciated, that the earlier one utilized rhythm control in patients with AF, the more likely it was to be effective in maintaining sinus rhythm. For example, Dittrich et al.^9^ demonstrated that if one cardioverted a patient with AF back to sinus rhythm, patients with a shorter history of AF tended to maintain sinus rhythm better than those with a longer history of AF. Also, as an example, if one compares both pivotal trials of sustained-release propafenone for AF,^10,11^ the same AAD in the same dose was more effective at maintaining sinus rhythm in the trial in which patients had a shorter history of AF and fewer major comorbidities. More recently, additional, much larger trials examining the effect of earlier AF rhythm control have added confirmatory and expanded information to these datasets.^12–17^ Because the data from these clinical trials and their implications for practice have already been reviewed extensively,^18,19^ I will not detail them here. Nonetheless, they appear to show that, with our current treatment options, earlier rhythm control versus more conventional management approaches are associated not only with a greater ability to maintain sinus rhythm^12^ but also potentially with improving cardiovascular outcomes, including cardiovascular death, stroke, and HF hospitalizations. Moreover, such an outcome benefit has been reported (1) in patients across the spectrum of left ventricular ejection fraction, (2) in patients with symptomatic as well as asymptomatic AF, (3) in both younger and older patients and in those with a greater comorbidity burden, and (4) in patients both with paroxysmal as well as persistent AF presentations.^13–17^ Finally, to a major degree, achieving sinus rhythm appears to be the key mediator of early rhythm control, leading to reduced cardiovascular complications. In the Early Treatment of Atrial Fibrillation for Stroke Prevention Trial 4 (EAST-AFNET 4), sinus rhythm at 12 months explained 81% of the treatment effect of early rhythm control compared to usual care during the remaining follow-up period of 4.1 years.^17^ Additionally, the benefits were seen not only in patients treated with ablation but also in those whose rhythm-control plan included AADs. In EAST-AFNET 4, only 8% of the early rhythm-control patients underwent ablation; 92% received treatment with an AAD, including medications from all AAD classes (with <20% receiving amiodarone). By 2 years, only 19.4% of the early rhythm-control arm had received ablation.^12^ Similarly, in a very large Korean trial of >50,000 subjects, most rhythm-control patients were treated with AADs rather than ablation.^16^

Thus, regardless of whether we define AF treatment efficacy by measurements of AF reduction (eg, lower AFB, lesser AF symptoms) or by a reduction of major adverse AF-related outcomes, they both appear to be related to more time in sinus rhythm. Likely, this relates to lesser adverse atrial remodeling when comorbidities are limited in number and progression and when the effects of recurrent and progressive AF on the atria are similarly inhibited. It is both the comorbidities and AF itself that lead to the development of and progression of the atrial myopathy (also termed atrial cardiomyopathy) that underlies worsened atrial function, impaired cardiac performance, AF progression, and thrombogenesis.^19–22^ This concept is discussed further by me in another paper in this same issue of the *Journal of Innovations in Cardiac Rhythm Management*.^23^ It then stands to reason that earlier intervention is more likely than later intervention both to restore sinus rhythm as well as to limit pathological atrial remodeling and, hence, the consequences (including symptoms and adverse outcomes) that come with it. Which endpoint to use, the reduction of AFB and symptoms, or the reduction of major adverse outcomes depends primarily on whether one is focusing on individual patients or on more global populational care. Consequently, efficacy measurements may be different in the individual patient (improved QoL) or in population studies (mortality, stroke, hospitalization patterns). So, the determination of efficacy is dependent upon the reason it is being sought. Importantly, this concept is valid whether the therapy is pharmacologic or ablative—or, as is often the case in the “real world,” a combination of both.

Finally, let us return to our patient from earlier. It has now been 2 years since she began her AAD—which has been well tolerated. On it, she still has occasional AF. However, the episodes have decreased to just 1–2 every 6 months and now last <30 min, with no symptoms except for a vague awareness of palpitations. No episode has required cardioversion. The patient is quite satisfied with this result and does not want to change drugs or pursue ablation. Thus, total elimination of the arrhythmia is not required to declare efficacy—treatment is not an exercise in perfection. With the individual patient, success is an adequate reduction of events such that the QoL is improved to their satisfaction, while assuring the fact that recurrences (under rate control and anticoagulation) do not pose any adverse hemodynamic or ischemic sequelae.
